# Risk and clinical-outcome indicators of delirium in an emergency department intermediate care unit (EDIMCU): an observational prospective study

**DOI:** 10.1186/1471-227X-13-2

**Published:** 2013-01-29

**Authors:** José Mariz, Nadine Correia Santos, Hugo Afonso, Pedro Rodrigues, António Faria, Nuno Sousa, Jorge Teixeira

**Affiliations:** 1 Life and Health Sciences Research Institute (ICVS), School of Health Sciences, University of Minho, Braga, Portugal; 2 ICVS/3B’s, PT Government Associate Laboratory, Braga/Guimarães, Portugal; 3 Clinical Academic Center – Braga, Braga, Portugal; 4 Emergency Department, Intermediate Care Unit (EDIMCU), Hospital de Braga, Braga, Portugal

**Keywords:** Emergency department, Intermediate care units, Short stay units, High dependency units, Delirium, Confusion assessment method, Length of stay, Osmolarity

## Abstract

**Background:**

Identification of delirium in emergency departments (ED) is often underestimated; within EDs, studies on delirium assessment and relation with patient outcome in Intermediate Care Units (IMCU) appear missing in European hospital settings. Here we aimed to determine delirium prevalence in an EDIMCU (Hospital de Braga, Braga, Portugal) and assessed routine biochemical parameters that might be delirium indicators.

**Methods:**

The study was prospective and observational. Sedation level was assessed via the Richmond Agitation-Sedation Scale and delirium status by the Confusion Assessment Method for the ICU. Information collected included age and gender, admission type, Charlson Comorbidity Index combined condition score (Charlson score), systemic inflammatory response syndrome criteria (SIRS), biochemical parameters (blood concentration of urea nitrogen, creatinine, hemoglobin, sodium and potassium, arterial blood gases, and other parameters as needed depending on clinical diagnosis) and EDIMCU length of stay (LOS). Statistical analyses were performed as appropriate to determine if baseline features differed between the ‘Delirium’ and ‘No Delirium’ groups. Multivariate logistic regression was performed to assess the effect of delirium on the 1-month outcome.

**Results:**

Inclusion and exclusion criteria were met in 283 patients; 238 were evaluated at 1-month for outcome follow-up after EDIMCU discharge (“good” recovery without complications requiring hospitalization or institutionalization; “poor” institutionalization in permanent care-units/assisted-living or death). Delirium was diagnosed in 20.1% patients and was significantly associated with longer EDIMCU LOS. At admission, Delirium patients were significantly older and had significantly higher blood urea, creatinine and osmolarity levels and significantly lower hemoglobin levels, when compared with No Delirium patients. Delirium was an independent predictor of increased EDIMCU LOS (odds ratio 3.65, 95% CI 1.97-6.75) and poor outcome at 1-month after discharge (odds ratio 3.51, CI 1.84-6.70), adjusted for age, gender, admission type, presence of SIRS criteria, Charlson score and osmolarity at admission.

**Conclusions:**

In an EDIMCU setting, delirium was associated with longer LOS and poor outcome at1-month post-discharge. Altogether, findings support the need for delirium screening and management in emergency settings.

## Backgrounds

As the acute diagnostic and treatment centers that provide a primary safety net with a 24/7 portal for rapid inpatient admission, modern emergency departments (ED) serve as a hub for emergency medical systems [1]. Within EDs, there is a rapid grow of Intermediate Care Units (IMCU) that are multi-purpose, high-dependency units [step-up from hospital wards and step-down from intensive care units (ICU)]. Patients admitted to high dependency units do not require full intensive care but need more services than those provided on a hospital ward [2,3], which calls for assiduous and rapid observation/intervention as the patient’s clinical condition evolves. The mean length of stay (LOS) in a standard EDIMCU is relatively short (24-72 hours) which may preclude/limit full information availability/assessment of the patient’s “normal functioning”. In this context, delirium may be a critical clinical factor to consider.

Delirium is defined as an acute change or fluctuation in mental status characterized by disorganized thinking and/or altered level of consciousness; importantly, it has a fluctuating course characterized by polymorphous and volatile symptoms [4]. Despite progress in the understanding of its clinical presentation, analysis of its clinical epidemiology, presentation and consequence to the overall clinical outcome remains complex [5-11]. In fact, although studies have indicated that delirium is a predictor of a longer hospital stay [5], there is limited work concerning delirium prevalence and physician detection rates in the emergency and/or acute care setting(s); furthermore, published data is predominantly from North America [9,12-14]. This gap in knowledge is especially critical given the differences in the breath (or management) of clinical-care provided in the emergency setting between the North American and European emergency systems and, consequently, its imprint on patient demographics [15]. Moreover, recent recommendations by the Society for Academic Emergency Medicine and by the American College of Emergency Physicians identified the detection of delirium in the ED as a high yield research objective [12]; nonetheless, although an increasing number of hospitals have created EDIMCUs, there are few data in the literature regarding delirium and outcomes in EDs and IMCUs [2,13] compared to the information in critically ill patients. In fact, with respect to delirium management, the few studies conducted in Europe included only 3% of the doctors working in high-dependency units [16]. This may be unrepresentative given the growing relevance of these units in emergency setting according to health policy reports [17].

Here, the main objective was to explore a relationship between delirium onset in an EDIMCU and patient outcome after discharge. For this, delirium occurrence among patients admitted to the EDIMCU at the Hospital de Braga (Braga, Portugal) was assessed and related with clinical and biochemical information/parameters that served to orient the criteria for EDIMCU admission/care, together with EDIMCU admission type and LOS. Delirium was assessed with the Confusion Assessment Method for the Intensive Care Unit (CAM-ICU) [9,14], given its ease of use, brevity and inter-rater reliability. Patient outcome was evaluated at 1-month after discharge.

## Methods

### EDIMCU

The study was conducted at the EDIMCU of the Hospital de Braga (Braga, Portugal), a University of Minho (Braga, Portugal) affiliated hospital (705-beds) that serves a population of 1,200,000 as a tertiary referral center. The Hospital de Braga has an ED with an annual census of approximately 175,000 visits; the ED and the EDIMCU are physically connected and the EDIMCU is part of the ED, sharing medical and nursing staff. The EDIMCU is a windowless 9-bed unit that receives patients from multiple intra and inter-hospital origin, including from the ED, surgical and medical wards (as a step-up unit), ICU (as a step-down unit), recovery operatory room, and other hospitals (without intermediate and/or intensive care units). The criteria for admission to the EDIMCU follow the Guidelines on Admission and Discharge for Adult Intermediate Care Units of the Society of Critical Medicine [18]. The unit provides non-invasive ventilation, invasive haemodynamic monitoring and inotrope infusion for high-risk medical and surgical patients; it does not provide renal replacement therapy or intracranial pressure monitoring. The standard nurse to patient ratio is 1:4 and a medical doctor is physically present in the unit (12-hour shifts).

### Patients and study design

During a four-month period in April 2012 to July 2012, data was prospectively collected on all consecutive admissions to the EDIMCU (Hospital de Braga, Braga). Inclusion criteria included: patients aged 18 years or older admitted to the EDIMCU for more than 24hrs. Patients were excluded from the final analysis if the clinical staff was unable to assess for delirium using the Confusion Assessment Method for the ICU (CAM-ICU) at any time during the admission, including due to clinical evaluation refusal by the patient, inability to follow simple commands before acute illness onset, language communication barriers, dementia or other diagnosed neuropsychiatric disorder and coma. Exclusion criteria followed that reported in similar studies [9,19]. The delirium assessment analysis was completed for all patients who met the inclusion criteria (n = 283). Patients were followed at day 30 after hospital discharge (1-month follow-up); electronic charts were reviewed to ascertain the status of the patients, and when no up-to-date information was available patients or caregivers were contacted by telephone in an open-way interview. Outcomes were recorded as either “good” (recovery without complications requiring hospitalization or institutionalization) or “poor” (institutionalization in permanent care-units/assisted-living or death). For this observational study the Ethical Committee at Hospital de Braga approved the study protocol and waived informed consent. The study was non-interventional; therapies with regard to the clinical diagnosis, delirium and sedation state were left to the discretion of each patient’s attending physician.

### Data collection and study design

Data were recorded prospectively at least once per 12-hour shift as part of the routine care, starting in the first 12 hours of admission to the EDIMCU. EDIMCU nursing staff assessed sedation level via the Richmond Agitation-Sedation Scale (RASS) and delirium status via the CAM-ICU (Portuguese translation available at [20]), following the same methodology reported by Han et al. [9,14]) (see Additional files 1 and 2). The CAM-ICU is a modified version of the Confusion Assessment Method (CAM) that objectively reports on: i) acute onset of mental status changes or a fluctuating course, ii) inattention, iii) disorganized thinking, and iv) altered level of consciousness [21]. Because it is easy and brief (less than 2 minutes) to administer, the CAM-ICU is also ideal for the ED environment. The CAM-ICU has high sensitivity (93% to 100%), specificity (98% to 100%) and excellent inter-rater reliability (κ = 0.77 to 0.95) [10]. In patients who were CAM-ICU positive, the Richmond Agitation and Sedation Scale (RASS) was used to categorize the psychomotor subtype of delirium (RASS score between: +1 and +4, hyperactive delirium; 0 and -3, hypoactive delirium; with both positive and negative scores at 0 and 3 hrs, mixed type) [22,23]. Before the start of data collection, all staff that participated in patient evaluation was part of a 4-month training period coordinated by two staff members (as part of the hospital Quality Assurance Program). Training materials were provided by Vanderbilt University and included training manuals, didactic lectures, demonstrations, and direct practice of the assessment tools in patient scenarios. Practical clinical vignettes were conducted by the ED nursing staff to check the inter-rater reliability of the RASS and CAM-ICU.

Patient information collected prospectively at the time of admission to the EDIMCU included: demographics (age and gender), admission diagnosis, Charlson Comorbidity Index combined condition and age-related score (which represents the sum of a weighted index that takes into account the number and seriousness of pre-existing co-morbid conditions [24], Charlson score), and blood parameters (including blood concentration of urea nitrogen, creatinine, hemoglobin, sodium and potassium, arterial blood gases, and other parameters as needed depending on clinical diagnosis; see Additional file 3). The EDIMCU protocol relies on blood analysis within 12 hours prior to admission. If patients do not have blood data in this time range and/or the clinical situation mandates prompt evaluation, blood parameters’ analysis is conducted immediately at EDIMCU admission; therefore, all the biochemical data presented falls within the 12-hours range prior to admission. The usual parameters that serve to orient the criteria for admission and care were considered regarding delirium occurrence; furthermore, information collected at discharge from the EDIMCU included the biochemical parameters considered at admission and that regarding the place to where the patient was released to (family/home or institution). The diagnostic categories for EDIMCU admission, assessed by the patients’ medical teams, represented the diagnostic category most representative of admission (cardiovascular, drug toxicity/withdrawal, gastrointestinal, genitourinary, neurologic, haemato-oncologic, pulmonary, trauma/musculoskeletal and other). Admission type (emergency department, operating room, wards, intensive care unit, inter-hospital transfer) was also recorded. The Charlson score, systemic inflammatory response syndrome (SIRS) criteria (two or more of the following criteria: heart rate > 90 beats ⁄ min; body temperature < 36 or > 38°C; respiratory rate > 20 breaths ⁄ min; white blood cell count < 4x10^9^ or > 12x10^9^ cells ⁄ L [25]) and biochemical parameters were used as a surrogate for severity of illness. The acute physiology and chronic health evaluation II (APACHE II) was not applied; the APACHE II has not been validated for EDs or IMCU and the constraints of the EDIMCU context (balance of amount of prospective data collected versus feasibility) was considered.

### Statistical analysis

Fisher’s exact tests, exact chi-square tests, independent-samples Mann Whitney, independent-samples *t*-test and one-way ANOVA analysis were performed as appropriate to determine if baseline features differed between ‘Delirium’ and ‘No Delirium’ groups. Multivariate logistic regression was performed to assess the effect of delirium on the 1-month outcome after discharge from the EDIMCU. Age, gender, admission type, presence of SIRS criteria, Charlson score and osmolarity at admission were considered covariates. All statistical analyses were conducted using PASW statistics version 18.0 (SPSS).

## Results

### Baseline characteristics and delirium clinical outcome

**Figure 1:**
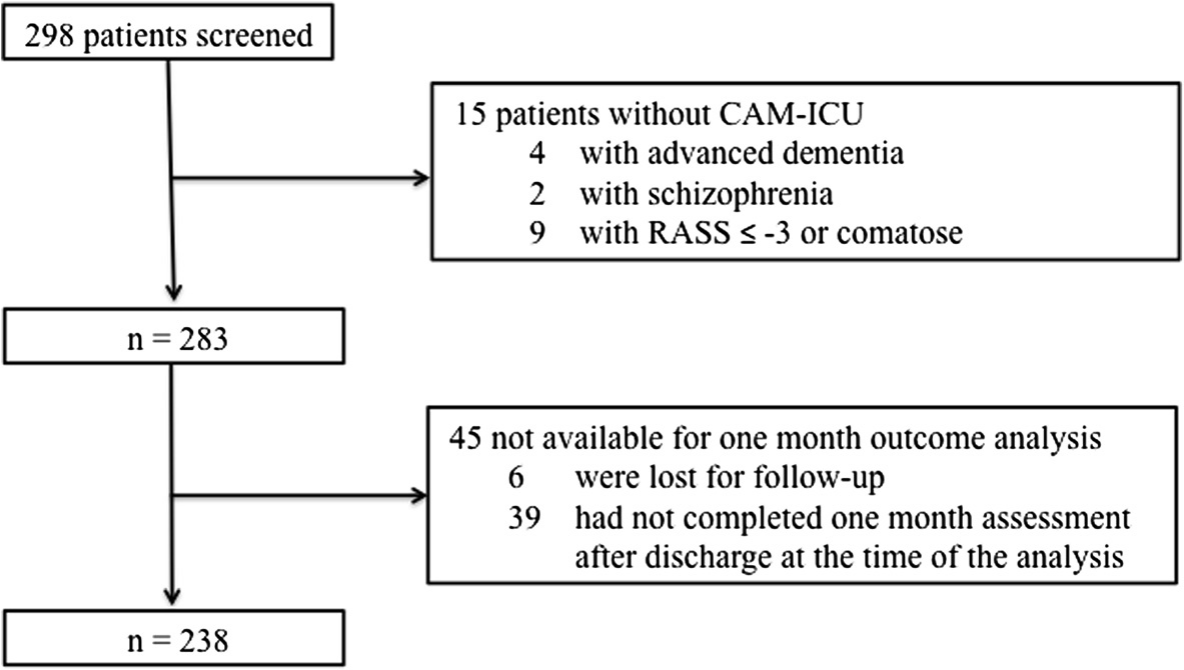
Patients meeting inclusion and exclusion criteria.

Of the 298 patients screened, 283 patients met the inclusion criteria and 15 were excluded (Figure 1). Patients were divided in two groups: ‘Delirium’ (n = 57, 20.1%) and ‘No Delirium’ (n = 226, 79.9%). Baseline characteristics are presented in Table 1. Thirty-nine of the delirium cases (68.4%) were detected in the first 24 hours of admission, 13 (22.8%) between 24 and 72 hours, and 5 (8.8%) after 72 hours from admission. Thirty-two cases of delirium (56.1%) had the duration of 1 day, 14 cases (24.6%) 2 days, 7 cases (12.3%) 3 days, 3 cases 4 days (5.3%) and 1 case 6 days.

**Table 1:** Patient demographics and characteristics stratified by delirium status

	**Delirium**	**No delirium**	**p-value**
**Patients, n (% total)**^a^	57 (20.1)	226 (79.9)	
**Characteristic**			
Mean age, years (SD)	67.1 (± 16.0)	60.3 (± 17.6)	0.010
Female, n (% group)	18 (31.6)	89 (39.4)	0.278
SIRS criteria, %	38.6	23.5	0.028
Median Charlson (IQR)	4 (5)	3 (4)	0.039
**Admission type (intra/inter hospital transfer category), n (% group; % origin)**^**b**^			< 0.001
Emergency department	28 (49.1; 23.0)	94 (41.6; 77.0)	
Operating room	6 (10.5; 6.4)	88 (38.9; 93.6)	
Wards	8 (14.0; 25.0)	24 (10.6; 75.0)	
Intensive care unit	7 (12.3; 63.6)	4 (1.8; 36.4)	
Inter-hospital transfer	8 (14.0; 33.3)	16 (7.1; 66.7)	
**Emergency physician diagnosis by organ system, n (% group; % diagnosis)**^**c**^			0.031
Cardiovascular	12 (21.1; 21.1)	45 (19.9; 78.9)	
Drug toxicity/withdrawal	2 (3.5; 33.3)	4 (1.8; 66.7)	
Gastrointestinal	11 (19.3; 11.7)	51 (22.6; 82.3)	
Genitourinary	6 (10.5; 22.2)	21 (9.3; 77.8)	
Neurologic	2 (3.5; 50.0)	2 (0.9; 50.0)	
Haemato-oncologic	4 (7.0; 11.8)	30 (13.3; 88.2)	
Pulmonary	13 (22.8; 36.1)	23 (10.2; 63.9)	
Trauma/musculoskeletal	7 (12.3; 21.9)	25 (11.1; 78.1)	
Other	0 (0.0; 0.0)	25 (11.1; 100.0)	

^a^Of the n = 298 patients enrolled, n = 15 were excluded due to dementia, neuropsychiatry illness or persistent coma (CAM-ICU assessment not conducted). ^b^Significant differences between ICU and ED (p = 0.008), OR (p < 0.001) and ward (p = 0.035), and between OR and ED (p = 0.016) and IHT (p = 0.020) (ANOVA, multiple comparisons Tukey post hoc HSD). ^c^Significant differences between Pulmonary and Other (p = 0.015) (ANOVA, multiple comparisons Tukey post hoc HSD). ED = emergency department, ICU = intensive care unit, IHT = inter-hospital transfer, IQR = interquartile range, OR = operating room; SD = standard deviation; SIRS = systemic inflammatory response syndrome.

Delirium patients were significantly older compared to No Delirium (mean 67.1 versus 60.2 years of age, p < 0.006), presented a higher percentage conforming to the SIRS criteria (38.6% versus 23.4%, p < 0.028) and had a higher Charlson score (median 4 versus 3, p < 0.039). Within the Delirium group no significant differences were noted regarding the time of delirium onset (after EDIMCU admission) and duration of delirium status; however, those with a mixed delirium subtype had a longer delirium status compared to hyper- and hypoactive delirium patients (p < 0.001) as well as a longer EDIMCU LOS between mixed and hypoactive (p < 0.009) (Table 2). No significant differences were noted between the Delirium and No Delirium groups regarding hospital LOS previous to EDIMCU admission; however, there was a significant difference between the Delirium and No Delirium groups for the EDIMCU LOS (median 1.0 versus 2.0 days, p < 0.001; odds ratio 3.65, 95% CI 1.97- 6.75). Significant differences were noted for type of hospital intra/inter transfer category prior to EDIMCU admission (p < 0.001); however, only ICU transfer (patients that were discharged from ICU and admitted in the EDIMCU) appeared as possible risk factor for delirium (63.6% Delirium versus 36.4% No Delirium) (Table 1). Regarding clinical status, cardiovascular, pulmonary, gastrointestinal, and haemato-oncologic were the most common reasons for admission to the EDIMCU; occurrence rates of delirium were significantly different between groups (p < 0.033), but only patients with neurologic-related diagnosis appeared more likely to develop delirium (equal percentage between those with Delirium versus No Delirium) (Table 1).

**Table 2:** Delirium status classified by delirium subtype

	**Hypoactive**	**Hyperactive**	**Mixed**
**Patients, n (% total)**	22 (38.6)	23 (40.3)	12 (21.0)
**Characteristic**			
Mean age, years (SD)	65.6 (± 17.3)	67.3 (± 15.6)	69.2 (± 15.3)
Female, n (% group)	9 (40.9)	6 (26.1)	3 (25.0)
Mean length of delirium, days (SD)^a^	1.2 (± 0.4)	1.6 (± 0.8)	3.0 (± 1.3)
Median EDIMCU LOS, days (IQR)^b^	2.0 (0.25)	2.0 (2.0)	4.0 (5.0)

^a^Significant differences between Mixed and Hypoactive (p < 0.001) and Hyperactive (p < 0.001) (ANOVA, multiple comparisons Tukey post hoc HSD). ^b^Significant differences between Mixed and Hypoactive (p = 0.009) (Kruskal-Wallis for median, K independent samples, independent samples median test). IQR = interquartile range; LOS = length of stay; SD = standard deviation.

### Biochemical parameters

For the analyzed biochemical parameters (see Additional file 3) at EDIMCU admission, when compared with No Delirium patients, Delirium patients had higher blood urea (mean 86.1 mg/dL versus 58.2 mg/dL, p < 0.001) and creatinine (mean 1.99 mg/dL versus 1.55 mg/dL, p < 0.006) at admission and lower hemoglobin concentration (mean 10.6 g/dL versus 11.3 g/dL, p < 0.038) (Table 3). Osmolarity and hemoglobin have a Pearson correlation value of 0.285 (p < 0.001). At discharge, delirium patients remained with significantly higher blood urea levels (mean 84.6 mg/dL versus 54.5 mg/dL, p < 0.006) and significantly lower hemoglobin concentrations (mean 10.0 g/dL versus 10.8 g/dL, p < 0.03) compared with No Delirium patients (Table 3). Osmolarity, a more accurate measure of (de)hydration than blood urea or sodium levels alone [26], was calculated from sodium, glucose and blood urea nitrogen levels at admission and was significantly different between groups (mean 320.55 mOsm/L versus 308.55 mOsm/L, p = 0.001). 

**Table 3:** Biochemical parameters stratified by delirium status

	**Delirium**	**No delirium**	**p-value**
**Biochemical parameters, mean (SD)**^**a**^			
Blood urea at admission, mg/dL	86.11 (67.57)	58.22 (50.97)	0.001
Blood urea at discharge, mg/dL	84.56 (70.15)	54.54 (45.25)	0.006
Hemoglobin at admission, g/dL	10.5 (2.7)	11.3 (2.6)	0.038
Hemoglobin at discharge, g/dL	10.0 (1.7)	10.8 (2.2)	0.030
Creatinine at admission, mg/dL	1.99 (1.66)	1.55 (1.56)	0.006
**Osmolarity, mOsm/L, mean (SD)**^**d**^	320.55 (23.20)	308.74 (17.37)	0.001

^a^Only statistical significant results are presented. Urea admission (Delirium, n = 57; No Delirium, n = 216); urea discharge (Delirium, n = 55; No Delirium, n = 212); hemoglobin admission (Delirium, n = 56; No Delirium, n = 221); hemoglobin discharge (Delirium, n = 56; No Delirium, n = 222); creatinine admission (Delirium, n = 57; No Delirium, n = 218). ^d^Plasma osmolarity measures the body's electrolyte-water balance [osmolarity = (2*sodium) + (glucose/18) + (blood urea nitrogen/3)], normal range 270-310 mOsm/L. Osmolarity calculated only for patients with all variable (sodium, glucose and blood urea) measurements at admission (Delirium, n = 48; No Delirium, n = 175). SD = standard deviation.

### One-month outcomes and multivariate analysis

At the 1-month outcome analysis 51 patients (17.1%) were excluded (patients with no contact and/or clinical information at 1-month after discharge); a total of 50 patients from the Delirium group and 188 from No Delirium group were evaluated (Figure 1 and Table 4). In the Delirium group mortality at the 1-month evaluation was 30% (combined death in the EDICUM and death after discharge; respectively, n = 7 and n = 8 for each setting) versus 10% for the No Delirium group (combined death in the EDICUM and death after discharge; respectively, n = 3 and n = 16) (p < 0.001). Furthermore, 26% patients were institutionalized versus 16.5% of the No Delirium group (p = 0.022). The estimated odds ratio for a poor outcome at 1-month associated with delirium status was 3.51 (CI 1.842 – 6.698). Delirium was independently associated with poor outcome at 1-month, defined as global mortality and/or institutionalization (multivariate logistic regression to assess the effect of delirium controlling for age, gender, admission type, SIRS criteria, Charlson score and osmolarity at admission).

**Table 4:** Clinical outcome and mortality at one-month assessment and multinomial logistical regression analysis results

**Outcome**	**Delirium**	**No delirium**	**Odds ratio (CI)**	**p-value**
**1-month, n (% group, % total)**^**a**^			3.51 (CI 1.84-6.70)	< 0.001
Good	22 (44.0; 9.2)	138 (73.4; 58.0)		< 0.001
Poor^b^	28 (56.0; 11.8)	50 (26.6; 21.0)		
**Poor, n (% group, % total)**^**b,c**^				
Death	15 (30.0; 19.2)	19 (10.1; 24.4)		< 0.001
Institutionalization	13 (26.0; 16.7)	31 (16.5; 39.7)		0.022
**EDIMCU LOS, days, median (IQR)**	2.0 (2.5)	1.0 (1.0)	3.65 (CI 1.97-6.75)	< 0.001
**Logistic regression parameter**	**Coeff. (SE)**		**Odds ratio (CI)**	**p-value**
Age	−0.00 (0.14)		1.00 (CI 0.97-1.02)	0.798
Gender	0.66 (0.64)		1.93 (CI 0.94-3.98)	0.075
Admission type	−0.31 (0.13)		0.57 (CI 0.57-0.95)	0.020
Charlson score	−0.08 (0.08)		0.93 (CI 0.79-1.08)	0.344
SIRS	−0.64 (0.38)		0.53 (CI 0.25-1.12)	0.095
Osmolarity	0.01 (0.01)		1.01 (CI 0.99-1.02)	0.602
Delirium	1.28 (0.40)		3.60 (CI 1.63-7.96)	0.002
Intercept	- 0.72 (3.03)			0.811

^a^Total sample n = 238, n = 6 patients were lost (no contact information or consult), n = 45 did not complete 1-month follow-up. ^b^Total poor outcome sample n = 78, poor outcome refers to mortality or institutionalization after discharge (permanent care units), with no significant difference between these. ^c^No significant difference in mortality between while in EDICUM or after discharge. *Charlson score* Charlson comorbidity index combined condition score; *CI* confidence interval (95% confidence interval for the odds ratio); *Coeff.*coefficient expressed in logits; *IQR* interquartile range; *LOS* length of stay; *SE* standard error; *SIRS* systemic inflammatory response syndrome criteria.

## Discussion

To our knowledge, this is the first report on delirium occurrence in a European EDIMCU. Results show 20.1% delirium prevalence (delirium patients significantly older than no delirium patients), with a significant relationship between delirium and mortality and LOS in the unit, and between delirium and global mortality and institutionalization at 1-month after discharge (all measures of poor outcomes). ICU transfer (at EDIMCU admission) appeared as a possible risk factor. Although not reaching statistical significance for delirium onset, it should be noted that 49.1% of the delirium patients were admitted from the ED (the ED and the EDIMCU are inter-supporting services at the Hospital de Braga and are physically bound in the same hospital wing), representing a total of approximately 1 in each 4 ED-origin patients developing delirium. The primary admission diagnosis and/or medical vs. surgical cases did not appear to impact delirium onset.

The significant positive relationship between delirium and EDIMCU LOS is in accordance with results of other studies conducted in EDs [7,27]; however, no significant difference in hospital LOS prior to EDIMCU admission was noted between delirium and non-delirium patients. The majority of delirium episodes occurred in the first 24 hour of admission, highlighting the importance of early screening in high-dependency units particularly, as was the case in this study, when a measure (information) on cognitive status prior to admission is not available. This observation is in line with other reports on delirium in the ED; it is advised screening in the first 12 hours of admission, to minimize extraneous factors that may artificially cause (new) onset delirium from prolonged exposure to known delirium precipitants (e.g. lack of windows, broken circadian rhythms with unscheduled admissions) [9]. Furthermore, our results indicate that screening should include assessment of routine biochemical parameters that may reflect dehydration, including blood urea, creatinine and osmolarity, as delirium indicators (these were significantly different between the Delirium and No Delirium groups). Results in these measures are more relevant in combination with the SIRS criteria and Charlson score; delirium patients presented significantly higher scores. Finally, multivariate analysis (controlling for age and gender, admission type, SIRS criteria, Charlson score and osmolarity at admission) significantly indicated that delirium status in the EDIMCU, independently of duration, relates with poor outcome at 1-month (that is, mortality or institutionalization in care-units).

Altogether, the results of the analysis are particularly relevant as the routine practice of delirium screening in the EDs remains limited and there are few data from the EDs and IMCUs literature regarding delirium and outcomes [2,13]. Here, the findings point to the main factors governing delirium in an acute setting: advanced age, admission type and dehydratation. As multicomponent strategies for the prevention of delirium have been developed for the hospital setting [28], it is unclear whether or not initiation of these interventions in the ED would improve outcomes. Of note, many of these multicomponent interventions require extensive resources and may not be feasible to perform in the ED setting. Nonetheless, some evidence indicates that increasing awareness of delirium through a brief and inexpensive education of staff on acute medical wards improves the rate of delirium detection [29,30]; this would be particularly optimal if associated with appropriate national guidelines and curriculums [29]. Therefore, simpler early detection-directed strategies focused on factors readily detectable by ED nursing and medical teams may probably be more effective than complex interventions requiring rigorous screening and specialized nursing [7,12,28]. Considering the substantial overlap between intermediate-care patients and less severely ill ICU patients [2], the rate detected in our cohort probably represents a continuum from severely ill to less severe patients. Of economic repercussion, the growing use of EDs, cited as a key contributor to rising health care costs, has become a leading target of health care reform [1]; therefore, the finding in EDIMCU that delirium is a predictor of longer LOS and mortality, and as well a predictor of greater level of dependency, is of particular relevance.

Critical care services vary between countries in both numbers of beds and volume of admissions, rendering in some cases distinction between intensive care and intermediate care units difficult [2,31,32]; importantly in the context of this study, is the fact that EDIMCU-type high-dependency units are much more common in Europe than in the US. The clinical features of high-dependency patients (as those in EDIMCU) are similar, but not identical, to those of less severely ill ICU monitor patients; therefore, comparisons should be adjusted for characteristics that previously have been shown to influence these outcomes [2]. Results of this cohort of high-dependency patients bounded to the ED require further analysis, particularly in comparison with non-ventilated ICU patients; however, routine daily delirium monitoring is already justified [5]. Ultimately, analysis of delirium rates and their outcome in the EDIMCU setting will help in the planning and debate over the roles and capabilities of this type of acute care areas.

## Conclusions

Delirium developed in approximately one fifth of the patients in the EDIMCU and was positively associated with age, longer EDIMCU LOS and poor outcome at 1-month after discharge (considering mortality and institutionalization, isolated and combined). Interestingly, although patients from ICU appeared to be at a greater delirium risk, delirium was not associated with the nature of admission. The main risk factors related with (de)hydration biochemical parameters.

### Key messages

- Studies on delirium prevalence and impact on patient outcome are missing in high-dependency EDIMCUs;

- Age, SIRS criteria, Charlson score and creatinine, blood urea, hemoglobin and osmolarity levels are indicators of delirium;

- Delirium is significantly associated with a longer EDIMCU LOS;

- Delirium is an independent risk factor of poor outcome (institutionalization and mortality) at 1-month after EDIMCU discharge;

- Further research is warranted to determine whether early detection alters outcome.

## Abbreviations

APACHE II: Acute physiology and chronic health evaluation II; CAM-ICU: Confusion assessment method for the intensive care unit; CI: Confidence interval; ED: Emergency department; EDIMCU: Emergency department intermediate care unit; ICU: Intensive care unit; IQR: Interquartile range; LOS: Length of stay; RASS: Richmond agitation-sedation scale; SD: Standard deviation; SIRS: Systemic inflammatory response syndrome.

## Competing interests

The authors declare that they have no competing interests.

## Authors’ contributions

Each author of this manuscript has: made substantial contributions to conception and design, acquisition of data, and the analysis or interpretation of data; been involved in drafting the article and revising it critically for important intellectual content; and given final approval of the submitted version to be published. All authors read and approved the final manuscript.

## Authors’ information

JM, Internist, Emergency Department, Emergency Department Intermediate Care Unit, Hospital de Braga, Braga and Doctoral researcher in neurosciences, Life and Health Sciences Research Institute (ICVS), School of Health Sciences, University of Minho, Braga, ICVS/3B’s, PT Government Associate Laboratory, Braga/Guimarães, Clinical Academic Center – Braga, Braga, Portugal; NCS, Post-doctoral researcher in neurosciences, Life and Health Sciences Research Institute (ICVS), School of Health Sciences, University of Minho, Braga, ICVS/3B’s, PT Government Associate Laboratory, Braga/Guimarães, Clinical Academic Center – Braga, Braga, Portugal; HA, Medical student, School of Health Sciences, University of Minho; PR, Nurse, Emergency Department, Hospital de Braga, Braga, Portugal; AF, Nursing Director, Emergency Department, Hospital de Braga, Braga, Portugal; NS, Full Professor in Medicine and Neurosciences, Life and Health Sciences Research Institute (ICVS), Vice-President, School of Health Sciences, University of Minho, Braga, ICVS/3B’s, PT Government Associate Laboratory, Braga/Guimarães, Clinical Academic Center – Braga, Director, Braga, Portugal; JT, Emergency Department Director, Emergency Department, Hospital de Braga, Braga, Portugal.

## Pre-publication history

The pre-publication history for this paper can be accessed here:

http://www.biomedcentral.com/1471-227X/13/2/prepub

## Supplementary Material

Additional file 1 A pdf file with the Authorized Portuguese translation of the CAM-ICU. Click here for file

Additional file 2 A pdf file with the EDIMCU clinical protocol. Click here for file

Additional file 3 A pdf file of the blood biochemical/clinical parameters at EDIMCU admission and discharge. Click here for file
