# Supplementary material for: Risk and clinical-outcome indicators of delirium in an emergency department intermediate care unit (EDIMCU): an observational prospective study
Source: BMC Emerg Med. 2013 Jan 29;13:2. doi: 10.1186/1471-227X-13-2 (PMC3563452; doi:10.1186/1471-227X-13-2)
Supplement: Additional file 1 — A pdf file with the Authorized Portuguese translation of the CAM-ICU. [file 1471-227X-13-2-S1.pdf]

The Confusion Assessment Method for the ICU
(CAM-ICU)

Training Manual

Este é um manual de treino para médicos, enfermeiros e outros profissionais de saúde que desejem usar o Confusion Assessment Method for the ICU (CAM-ICU).

O CAM-ICU é um instrumento de monitorização para doentes das UCIs. Este manual de treino fornece uma explicação detalhada de como usar o CAM-ICU, bem assim como respostas a perguntas feitas frequentemente.

Todas as perguntas devem ser dirigidas a:

E. Wesley Ely, MD, MPH, FACP, FCCP
Brenda Truman, RN, MSN, ACNP
Vanderbilt University Medical Center
brenda.truman@vanderbilt.edu
wes.ely@vanderbilt.edu

Cristina Granja, M.D.
Department of Intensive Care
Hospital Pedro Hispano, Matosinhos
Department of Biostatistics, Faculty of Medicine of Oporto, Portugal
Email: cristina.granja@hph.min-saude.pt

Alice Lopes, M.D.
Department of Psychiatry
Hospital Geral de Sto António, Oporto, Portugal

Copyright © 2002, E. Wesley Ely, MD, MPH and Vanderbilt University, all rights reserved

Introdução ao Manual de Treino do Método de Avaliação da Confusão (CAM-ICU)

Os nossos estudos prévios efectuados em doentes em ventilação mecânica (e.g., Ely, *N Engl J Med 1996*) e, em particular, aqueles dois estudos efectuados em doentes idosos com insuficiência respiratória (Ely, *Ann Intern Med 1999 and 2002*), ajudaram a direccionar a nossa atenção para o delírio/disfunção cognitiva aguda como uma área importante de foco na melhoria do tratamento do doente. A incidência de insuficiência respiratória aumenta cerca de 10 vezes à medida que a idade do doente aumenta dos 55 para os 85 anos de idade (Behrendt, *Chest 2000*). Em 2001, foi relatado que cerca de 2/3 de todos os dias de internamento nas UCIs eram ocupados por doentes com idade superior a 65 anos. (Angus, *JAMA 2001*). Se tivermos em conta os problemas particulares dos doentes idosos, quando conectados a próteses ventilatórias, o delírio e outras formas de disfunção cognitiva parecem-nos alcançar uma alta prioridade. De acordo com o National Research Council, "para muitas pessoas em boa condição física que sucumbem a uma doença aguda, o declínio cognitivo é a ameaça principal à sua capacidade de recuperação e de apreciar as suas actividades favoritas; para aqueles cuja actividade física era já limitada, o declínio cognitivo é uma ameaça adicional à qualidade de vida" (The Aging Mind, National Academy Press 2000).

Começamos por construir um programa de investigação do delírio na UCI com vista a estudar a incidência e a importância prognóstica do delírio nos doentes de todas as idades ventilados mecanicamente. Ao pesquisar na literatura um instrumento validado para ser usado em doentes ventilados, ficamos surpresos por verificar que na metodologia de todos os estudos sobre delírio, encontrávamos sempre a mesma frase: "Os doentes ventilados mecanicamente foram excluídos". Na sequência destes achados, iniciamos uma colaboração internacional com peritos multidisciplinares na área do delírio, num esforço para desenvolver um instrumento apropriado para doentes nas UCIs, ventilados ou não.
O instrumento mais largamente disseminado para avaliaçãod o delírio por não-psiquiatras era o Método de avaliação da confusão - Confusion Assessment Method ou CAM (Inouye, *Ann Intern Med 1990*). Por esta razão decidimos adaptar este instrumento e trabalhamos com a Dra Sharon Inouye, de Yale, para adaptar e validar o CAM-ICU.

Este manual de treino é o resultado do trabalho realizado entre 1998 e 2003. Incluímos uma página de bibliografia, a qual inclui dois artigos de revisão sobre o delírio, um artigo que descreve o resultado (outcome) associado com o delírio na UCI, os dois estudos originais de validação do CAM-ICU, os dois estudos originais da escala de agitação e sedação de Richmond (Richmond Agitation Sedation Scale), e as guidelines para prática clínica da Sociedade de Medicina Crítica (Society of Critical Care Medicine) para a analgesia e sedação, emitidas em 2002.

Ao continuarmos com os nossos estudos de validação, acreditamos que este instrumento pode fornecer uma ferramenta que pode ser usada por enfermeiros, médicos, ou qualquer outro profissional de saúde dentro da equipa multidisciplinar da UCI. O CAM-ICU está a ser usado de forma regular num número crescente de UCIs como parte da avaliação clínica de rotina e foi escolhida para numerosos estudos de investigação que estão a decorrer em mais de sete países. È nossa esperança que através do seu uso clínico e através dos estudos a decorrer, o prognóstico (outcome) dos doentes possa melhorar – o nosso objectivo final!

A nossa equipa tem muito gosto em ajudar a responder a qualquer questão que possa enfrentar, enquanto implementa o CAM-ICU. Todos os materiais estão disponíveis electronicamente, desde que requisitados. Nós actualizamos frequentemente este manual de treino, pelo que apreciamos qualquer feedback. Por favor, sinta-se à vontade para nos telefonar ou contactar via e-mail a propósito de qualquer erro ou comentário que considere pertinente em relação com o CAM-ICU ou este manual de treino.

Atenciosamente,

E. Wesley Ely, MD, MPH, FACP, FCCP Vanderbilt University Medical Center
Brenda Truman, RN, MSN, ACNP

Bibliografia

Delirium Overviews

Ely, E.W., Siegel, M.D., Inouye, S.K. Delirium in the intensive care unit: An under-recognized syndrome of organ dysfunction. Semin Respir Crit Care Med 2001; 22:115-126.

Truman B., Ely E.W. Monitoring delirium in critically ill patients. Crit Care Nurse 2003; 23:25-36.

Ely, E.W., Gautam, S., Margolin, R., Francis, J., May, L., Speroff, T., Truman, B., Dittus, R., Bernard, G.R., Inouye, SK. The impact of delirium in the intensive care unit on hospital length of stay. Intensive Care Med 2001; 27:1892-1900.

CAM-ICU Validation Studies

Ely, E.W., Inouye, S., Bernard G., Gordon, S., Francis, J., May, L., Truman, B., Speroff, T., Gautam, S., Margolin, R, Dittus, R. Delirium in mechanically ventilated patients: validity and reliability of the confusion assessment method for the intensive care unit (CAM-ICU). JAMA 2001; 286: 2703-2710.

Ely, E.W., Margolin, R., Francis, J., May, L., Truman, B., Dittus, B., Speroff, T., Gautam, S., Bernard, G., Inouye, S. Evaluation of delirium in critically ill patients: Validation of the Confusion Assessment Method for the Intensive Care Unit (CAM-ICU). Crit Care Med 2001; 29:1370-1379.

RASS Validation Studies

Sessler, C.N., Gosnell, M., Grap, M.J., Brophy, G.T., O'Neal, P.V., Keane, K.A., Tesoro, E.P., Elswick, R.K.. The Richmond Agitation-Sedation Scale: validity and reliability in adult intensive care patients. Am J Respir Crit Care Med 2002; 166:1338-1344.

Ely, E.W., Truman, B., Shintani, A., Thomason, J.W.W., Wheeler, A.P., Gordon, S., Francis, J., Speroff, T., Gautam, S., Margolin, R., Dittus, R., Bernard, G., Sessler, C.N.. Monitoring sedation status over time in ICU patients: the reliability and validity of the

Richmond Agitation Sedation Scale (RASS). JAMA 2003; 289:2983-2991.

Clinical Practice Guidelines

Jacobi, J., Fraser, G.L., Coursin, D.B., Riker ,R., Fontaine, D., Wittbrodt, E.T., Chalfin, D.B., Masica, M.F., Bjerke, S., Coplin, W.M., Crippen, D.W., Fuchs, B.D., Kelleher, R.M., Marik, P.E., Nasraway, S.A., Murray, M.J., Peruzzi, W.T., Lumb, P.D.. Clinical practice guidelines for the sustained use of sedatives and analgesics in the critically ill adult. Crit Care Med 2002; 30:119-141.

Conectar a Monitorização da Sedação e Do Delírio:
Um Método de Dois Passos para Avaliar a Consciência

Passo um: Avaliação da Sedação

A Escala de Agitação e Sedação de Richmond: a RASS*
(The Richmond Agitation and Sedation Scale: The RASS*)

Pontuação	Termo	Descrição	
+4	Combativo	Abertamente combativo, violento, representa perigo imediato para o pessoal da UCI	
+3	Muito agitado	Puxa ou retira tubos ou catéteres; agressivo	
+2	Agitado	Movimentos não intencionais frequentes; luta contra o ventilador	
+1	Inquieto	Ansioso mas os movimentos não são agressivos	
0	Desperto e calmo		
-1	Confuso	Não está completamente desperto, mas consegue manter-se acordado (abertura dos olhos/contacto dos olhos) *à voz* **(>10 segundos)**	**Estimulação Verbal**
-2	Sedação Ligeira	Acorda por breves períodos com contacto dos olhos *à voz* **(<10 segundos)**	
-3	Sedacão Moderada	Movimento ou abertura de olhos *à voz* (mas sem contacto dos olhos**)**	
-4	Sedação Profunda	Não responde à voz, mas movimentos ou abertura dos olhos com *estímulo táctil*	**Estimulação Física**
-5	Não despertável	Não responde à *voz* ou *ao estímulo táctil*	

Se RASS for -4 ou -5, **Parar** e **Reavaliar** o doente mais tarde

Se RASS for superior a - 4 (-3 até +4) então **Seguir para o Passo 2**

*Sessler, et al. AJRCCM 2002; 166:1338-1344.

*Ely, et al. JAMA 2003; 289:2983-2991.

Passo Dois: Avaliação do Delírio

Característica 1: Início agudo de alterações do estado mental ou curso fluctuante

e

Característica 2: Falta de Atenção

e

Característica 3: Pensamento desorganizado OU **Característica 4**: Nível de Consciência alterado

= DELIRIO

5

CAM-ICU – Características e Descrições

1. Início agudo ou curso flutuante **Ausente** **Presente**

A. Há evidência de uma alteração aguda no estado mental em relação ao estado basal?

OU

B. Este comportamento (anormal) flutuou nas últimas 24 horas, isto é, teve tendência a ir e vir, ou aumentar ou diminuir na sua gravidade, tendo sido evidenciado por flutuações na escala de sedação (e.g., RASS), Glasgow, ou avaliação de delírio prévia?

2. Falta de Atenção **Ausente** **Presente**

O doente teve dificuldades em focar a atenção, tal como evidenciado por índices inferiores a 8 quer no componente visual quer no componente auditivo do **Teste de Atenção Attention Screening Examination (ASE)** ? (Instruções na página seguinte).

3. Pensamento desorganizado **Ausente** **Presente**

Existem sinais de pensamento desorganizado ou incoerente tal como evidenciado por respostas incorrectas a duas ou mais das 4 questões e/ou incapacidade de obedecer aos seguintes comandos:

Questões (Alternar Conjunto A e Conjunto B)**:**

**Conjunto A**	**Conjunto B**
1. Uma pedra pode flutuar na água?	1. Uma folha pode flutuar na água?
2. Existem peixes no mar?	2. Existem elefantes no mar?
3. Um quilo pesa mais do que dois quilos?	3. Dois quilos pesam mais do que um quilo?
4. Pode usar-se um martelo para pesar uma agulha ?	4. Pode usar-se um martelo para cortar madeira?

Outras:

1. Está com o seu pensamento pouco claro?
2. Segure nestes dedos. (O examinador coloca dois dedos em frente do doente)
3. Agora faça o mesmo com a outra mão. (Não repetir o número de dedos)

4. Nível de consciência alterado **Ausente** **Presente**

O nível de consciência do doente é outro qualquer que *não o alerta*, tal como o vigil, letárgico ou estuporoso ? (i.e., RASS diferente de “0” na altura da avaliação)

Alerta completamente ciente do ambiente, e interactua apropriadamente de forma

espontânea

Vigilante hiperalerta

Letárgico sonolento mas facilmente despertável, não ciente de alguns elementos do ambiente ou não interactua de forma apropriada com o entrevistador; torna-se completamente ciente do ambiente e interactua apropriadamente quando estimulado minimamente

Estuporoso completamente alheado mesmo quando estimulado vigorosamente; só despertável com estímulos vigorosos e repetidos, e assim que o estímulo cessa, o indivíduo estuporoso volta para o estado anterior de não despertável

CAM-ICU Global (Características 1 e 2 e quer característica 3 ou 4): **Sim** **Não**

Teste de Atenção (The Attention Screening Examination (ASE) – auditivo e visual

A. Teste de Atenção Auditivo (Letras)

Orientações: Diga ao doente: "Vou ler-lhe uma série de 10 letras. Sempre que ouvir a letra "A", indique-me apertando a minha mão." Leia as seguintes 10 letras num tom de voz normal (suficientemente alto para ser ouvido acima do ruído da UCI) à velocidade de uma letra por segundo.

S A H E V A A R A T

Pontuação: Os erros são contados quando o doente falha no aperto de mão aquando da letra "A" e quando o doente aperta a mão em qualquer outra letra que não a "A".

B. Teste de AtençãoVisual (Figuras)

*** * Ver o seguinte conjunto de figuras (A and B) * ***

Passo 1: 5 figuras

Orientações: Diga ao doente, "Sr. ou Sra. _________________, vou mostrar-lhe figuras que representam alguns objectos comuns. Observe-os com atenção e tente recordar-se de cada figura porque vou perguntar-lhe quais foram as figuras que viu. A seguir mostre o passo 1 quer do conjunto A quer do conjunto B, alternando diariamente se forem feitas várias medições. Mostre as primeiras 5 figuras durante 3 segundos cada uma.

Passo 2: 10 figuras

Orientações: Diga ao doente, "Agora vou mostrar-lhe mais algumas figuras. Algumas delas já as viu e algumas são novas. Indique se já viu ou não estas fotografias abanando a cabeça para sim (demonstrar) ou não (demonstrar). A seguir mostre 10 figuras (5 novas e 5 repetidas) durante 3 segundos cada.

(Passo 2 do conjunto A ou B, dependendo do que foi usado no passo 1 anterior).

Pontuação: este teste é pontuado pelo número de respostas "sim" ou "não" correctas durante o segundo passo (dentro de 10 possíveis). Com vista a melhorar a visibilidade para os doentes mais idosos, as imagens são imprimidas em tamanho 6x10 em papel colorido e

laminadas com acabamento mate.

Nota: Se o doente usa óculos assegurar que ele os tem colocado aquando da realização do teste visual.

Bibliografia:

Ely, E.W., Inouye, S., Bernard G., Gordon, S., Francis, J., May, L., Truman, B., Speroff, T., Gautam, S., Margolin, R, Dittus, R. Delirium in mechanically ventilated patients: validity and reliability of the confusion assessment method for the intensive care unit (CAM-ICU). JAMA; 286, 2703-2710, 2001.

Ely, E.W., Margolin, R., Francis, J., May, L., Truman, B., Dittus, B., Speroff, T., Gautam, S., Bernard, G., Inouye, S. Evaluation of delirium in critically ill patients: Validation of the Confusion Assessment Method for the Intensive Care Unit (CAM-ICU). Critical Care Medicine. 29:1370-1379, 2001.

Visual ASE - Packet A

Step 1

Visual ASE - Packet A

Step 2

Visual ASE - Packet B

Step 1

Visual ASE - Packet B

Step 2

Dúvidas Frequentes

Gerais

1. É possível aplicar a avaliação CAM-ICU em doentes dementes?

Vários graus de demência basal podem estar presentes nos nossos doentes, sem que tal tenha sido previamente reconhecido. Por esta razão é útil saber as características do delírio que podem ser diagnosticáveis mesmo na presença de demência (Trzepacz, *Journal of Neuropsychiatry 1998*.). De facto, nós efectuamos a avaliação do CAM-ICU em subgrupos de doentes com demência provável retirados dos estudos de validação (tal como fez o Dr Inouye no seu estudo de validação original).

O CAM-ICU foi fiável e válido em doentes quer com quer sem demência. Estes doentes apresentam, contudo, uma maior dificuldade na sua avaliação. Tanto quanto possível é importante identificar correctamente o estado basal cognitivo funcional e diferenciar perturbações cognitivas crónicas devidas a demência das alterações agudas na atenção e no pensamento devidas ao delírio. Nós rastreamos todos os doentes sob estudo com instrumentos de avaliação substitutos para a demência. [i.e. a versão modificada da Blessed Dementia Rating Scale or mBDRS (Blessed, *Brit.J.Psychiat 1968*) ou o Informant Questionnaire on Cognitive Decline in the Elderly or IQCODE (Jorm, *Psychological Medicine 1989*)].

As definições seguintes podem ajudar a sublinhar as características major que distinguem o delírio da demência:

Delírio: Um distúrbio da consciência caracterizado por início agudo e curso flutuante da função cognitiva alterada, de forma a que a capacidade do doente receber, processar, armazenar e recordar informações está marcadamente alterada. O delírio desenvolve-se ao longo de um curto període de tempo (horas ou dias), é geralmente reversível, e é consequência directa de uma condição médica, síndrome de intoxicação ou abstinência, medicação, exposição a toxinas, ou a combinação destes factores. Raciocínio: início rápido, consciência turva, com agravamento nocturno, flutuante.

Demência: Desenvolvimento de um estado generalizado de défices cognitivos no qual há

deterioração de capacidades intelectuais adquiridas previamente, cuja instalação ocorre, geralmente, ao longo de semanas ou meses. Os défices incluem alteração da memória e pelo menos um dos seguintes: afasia, apraxia, agnosia, ou distúrbio na função executiva. Os doentes com demência geralmente não exibem falta de atenção a não ser tardiamente no decurso da doença. Os défices cognitivos têm de ser suficientemente graves para provocarem alterações no funcionamento ocupacional e social, e podem ser progressivos, estáticos ou reversíveis dependendo da patologia e da disponibilidade do tratamento. Raciocínio: início gradual, diminuição da capacidade intelectual, distúrbios da memória, mudança na personalidade/humor, sem turvação da consciência.

2. Como é que se identifica o delírio num doente com afecto embotado secundário a uma depressão major?

Os doentes deprimidos podem exibir as características de delírio e são avaliáveis usando o CAM-ICU. Em casos raros, a depressão pode manifestar-se de forma a provocar um CAM-ICU falso positivo. No geral, este tipo de distinção deve incluir a opinião de um psiquiatra. Na maior parte dos casos, um doente deprimido com um CAM-ICU positivo é considerado como tendo delírio.

3. Junto ao leito do doente é necessário realizar a avaliação das 4 características em sequência?

Quando se pensa implementar o CAM-ICU quer na prática clínica, quer para fins de investigação, é importante considerar que muitos dos seus componentes são similares aos métodos menos formais de avaliação junto ao leito que são frequentemente usados na prática (i.e., quer os médicos, quer os enfermeiros ou outro pessoal de saúde, geralmente avaliam a característica 1 através de escalas de sedação ou avaliações neurológicas frequentes).

Uma avaliação cuidadosa dos componentes correntes de avaliação junto ao leito ajudam a

identificar quais as características do CAM-ICU que já estão a ser avaliadas.
Um exame das nossas práticas correntes na UCI pode ajudar também a modificar algumas partes da avaliação corrente de forma a definir o delírio com maior precisão. Recomendamos a inclusão das ferramentas do CAM-ICU na avaliação diária junto ao leito do doente. Os dados são colhidos ao longo da avaliação do doente e posteriormente são juntos no algoritmo do CAM-ICU com vista a discernir a presença ou ausência de delírio.

CARACTERÍSTICA 1: Início agudo ou flutuações no estado mental

1. Usa sempre o mesmo "estado basal" em avaliações CAM-ICU sucessivas?
Sim.

2. Como é que faz /faria se o doente tiver uma alteração permanente do seu estado basal durante a hospitalização, e.g., um acidente vascular cerebral? Esse novo estado basal torna-se o novo para os fins de CAM-ICU?

Se o doente tiver uma alteração permanente no seu estado basal (e.g., acidente vascular cerebral) então esse novo estado passa a ser o usado para todas as avaliações subsequentes do CAM-ICU. Contudo, determinar o estado basal pode ser difícil, em virtude da dificuldade inerente em separar delírio desse novo estado basal. Na prática, é mais fácil preencher a característica 1 em tal situação através da documentação de "flutuações" no estado mental.

Característica 2: Falta de atenção

O estado de alerta é um processo básico do estado desperto no qual o doente acordado pode responder a qualquer estímulo do ambiente.
O doente alerta, mas não atento, responde a qualquer som, movimento ou acontecimento na

sua proximidade, enquanto que o doente atento pode rejeitar estímulos irrelevantes.
A atenção pressupõe estar alerta, mas estar alerta não pressupões necessariamente estar atento (***i.e., todos os doentes atentos estão alerta, mas nem todos os doentes alerta estão atentos)*** (Strub, The mental status examination in neurology, F.A. Davis Company, 1993).

1. Como é que se distingue se a incapacidade para compreender as instruções é devida a falta de atenção ou a raciocínio desorganizado e incapacidade para compreender as instruções numa tentativa de distinguir sintomas de demência do delírio?

No início da avaliação da falta de atenção, o entrevistador estabelece se o doente é capaz ou não de seguir o mais simples "sim" ou "não" com movimento da cabeça ou apertar de mãos. Se o doente é capaz, então os dados são registados de acordo com o número de respostas correctas para o teste de figuras e letras da avaliação da atenção (ASE). Se um doente for capaz de efectuar mesmo as instruções mais básicas, então considera-se positivo para a característica 2 (i.e., não atento) se ele/ela somar menos de 8 respostas correctas no teste de avaliação visual (ASE). É correcto que possa existir também um elemento de desorganização o qual deve ser avaliado na característica 3.

2.
Quando os doentes estão muito letárgicos, estuporosos ou comatosos, os componentes do teste de avaliação da atenção (ASE) podem ser impossíveis de administrar. Se não se puder aplicar o teste, qual é a conclusão acerca do delírio – delírio?-não delírio?-não aplicável?

A abordagem de dois passos do CAM-ICU fornece um filtro para a maior parte dos doentes que não consegue comunicar com o avaliador. Os doentes que não progridem para o passo 2 (i.e., aqueles com nível de sedação RASS 4 a 5) não são testados com o restante da avaliação CAM-ICU. Por isso, para aqueles que atingem o passo 2 e apresentam abertura dos olhos apenas com estimulação verbal, a incapacidade para realizar ou completar os componentes do teste de atenção (ASE) é atribuída a falta de atenção. Estes doentes não são

capazes de atentar nos seus pensamentos (seja qual for a razão).

Uma pontuação RASS de –3 parece ser uma zona cinzenta. Alguns doentes neste estado conseguem comunicar enquanto que outros apenas abrem os olhos com uma interacção mínima. Colocámos o cut-off para os dois passos RASS entre –3 e –4 porque alguns doentes com RASS –3 conseguem ser avaliados capazmente.

CARACTERÍSTICA 3: Pensamento desorganizado

Esta é sem dúvida a área mais difícil de ser avaliada em doentes que não verbalizam. Esta é a mais subjectiva de todas as quatro características. O pensamento é expresso através de palavras (verbalizado ou escrito). A ventilação mecânica e a perda de capacidade para executar movimentos finos limitam a capacidade expressiva da maior parte dos doentes das UCIs. Como consequência destes factos, o CAM-ICU usa questões directas e fáceis de sim/não e comandos simples para avaliar a organização do pensamento. Estamos receptivos a melhorar os nossos métodos de avaliação desta característica do delírio, pelo que agradecemos o seu feedback nesta Característica.

1. Se um doente responder correctamente às 4 questões, avalia-se mesmo assim os comandos?

Encorajamos aqueles que aplicam o CAM-ICU a perguntar todas as questões e todos os comandos. Desencorajamos omitir os comandos (mesmo quando o doente tiver uma pontuação de 100%) em virtude da hipótese do doente ter acertado quatro questões por mero acaso.

A combinação das questões e dos comandos dá ao médico mais dados sobre os quais pode efectuar um julgamento acerca da presença ou ausência de pensamento desorganizado.

Se o doente responde correctamente a todas as questões, mas o avaliador sente que o doente acertou as perguntas por acaso, a capacidade de resposta nos comandos pode ajudar a confirmar ou não o palpite do médico.

NOTA: Os critérios para esta característica foram incorrectamente listados nas nossas publicações (Ely, et al. JAMA 2001; 286: 2703-2710. and Truman, et al CCN 2003; 23:25-36.). O pensamento organizado é evidenciado por 3 ou mais respostas correctas às 4 questões. Por esta razão (tal como listado na página 5 deste manual) os doentes pontuam

uma característica 3 positiva (i.e., pensamento desorganizado) quando respondem incorrectamente a duas ou mais das quatro questões.

CARACTERÍSTICA 4: Nível alterado de consciência (na altura da avaliação)

1. A característica 4 é positiva para o coma?

Embora os doentes comatosos sejam tecnicamente "CAM+", o coma não é considerado delírio. Contudo, um doente com delírio pode ter estado recentemente em coma, indicando uma flutuação no estado mental. Muitas vezes, embora não sempre, os doentes comatosos, progridem através de um período de delírio antes de recuperarem o seu estado mental de base. A característica 4 é positiva para qualquer doente com nível "RASS" diferente de "0".

Exemplos

**Característica CAM-ICU**	**Presente/Ausente**
**Característica 1: Início agudo de alterações no estado mental ou curso flutuante**	
O RASS do doente é no momento de "0", mas nas 24 h anteriores foi de –1, -3, e +2	Presente
O RASS do doente tem sido –2 nas últimas 24 h, mas a família atesta que esse não é o seu estado de base	Presente
**Característica 2: Falta de atenção**	
O doente pontuou 7 no teste de atenção (ASE) das figuras e 5 no das cartas	Presente
Um doente é capaz de responder correctamente a 10 perguntas do teste de atenção (ASE) quer nas figuras quer nas cartas	Ausente
O doente é capaz de comunicar através do aperto de mão do entrevistador, mas é incapaz de completar o teste ASE (figuras e cartas).	Presente
**Característica 3: Pensamento desorganizado**	
O doente responde correctamente apenas a metade das questões.	Presente
O doente responde correctamente a todas as perguntas e consegue identificar os dedos que o entrevistador expõe.	Ausente

Característica 4: Nível de consciência alterado

O doente apresenta frequentemente movimentos não intencionais e luta contra o ventilador (i.e., RASS=+2) Presente

O doente tem tido flutuações no estado mental, com diversas pontuações RASS nas últimas 24 h, mas agora está desperto e calmo (i.e., RASS=0) Ausente
