# Supplementary material for: Risk and clinical-outcome indicators of delirium in an emergency department intermediate care unit (EDIMCU): an observational prospective study
Source: BMC Emerg Med. 2013 Jan 29;13:2. doi: 10.1186/1471-227X-13-2 (PMC3563452; doi:10.1186/1471-227X-13-2)
Supplement: Additional file 3 — A pdf file of the blood biochemical/clinical parameters at EDIMCU admission and discharge. [file 1471-227X-13-2-S3.pdf]

Hypothesis Test Summary

	Null Hypothesis	Test	Sig.	Decision
1	The distribution of Hemoglobin concentration at admission g/dl is the same across categories of Delirium.	Independent-Samples Mann-Whitney U Test	.038	Reject the null hypothesis.
2	The distribution of Hemoglobin concentration at discharge g/dl is the same across categories of Delirium.	Independent-Samples Mann-Whitney U Test	.030	Reject the null hypothesis.
3	The distribution of Hematocrit at admission is the same across categories of Delirium.	Independent-Samples Mann-Whitney U Test	.125	Retain the null hypothesis.
4	The distribution of Hematocrit at admission is the same across categories of Delirium.	Independent-Samples Mann-Whitney U Test	.180	Retain the null hypothesis.
5	The distribution of Total white blood cell count at admission is the same across categories of Delirium.	Independent-Samples Mann-Whitney U Test	.749	Retain the null hypothesis.
6	The distribution of Total white blood cell count at discharge is the same across categories of Delirium.	Independent-Samples Mann-Whitney U Test	.844	Retain the null hypothesis.
7	The distribution of C-Reactive Protein at admission is the same across categories of Delirium.	Independent-Samples Mann-Whitney U Test	.089	Retain the null hypothesis.
8	The distribution of C-Reactive Protein at discharge is the same across categories of Delirium.	Independent-Samples Mann-Whitney U Test	.374	Retain the null hypothesis.
9	The distribution of Sodium concentration at admission is the same across categories of Delirium.	Independent-Samples Mann-Whitney U Test	.050	Retain the null hypothesis.
10	The distribution of Sodium concentration at discharge is the same across categories of Delirium.	Independent-Samples Mann-Whitney U Test	.132	Retain the null hypothesis.

Asymptotic significances are displayed. The significance level is .05.

[1] Exact significance is displayed for this test.

Hypothesis Test Summary

	Null Hypothesis	Test	Sig.	Decision
11	The distribution of Potassium at admission is the same across categories of Delirium.	Independent-Samples Mann-Whitney U Test	.247	Retain the null hypothesis.
12	The distribution of Potassium at discharge is the same across categories of Delirium.	Independent-Samples Mann-Whitney U Test	.803	Retain the null hypothesis.
13	The distribution of Aspartate aminotransferase at admission is the same across categories of Delirium.	Independent-Samples Mann-Whitney U Test	.162	Retain the null hypothesis.
14	The distribution of Alanine aminotransferase at admission is the same across categories of Delirium.	Independent-Samples Mann-Whitney U Test	.811	Retain the null hypothesis.
15	The distribution of Total bilirrubin at admission is the same across categories of Delirium.	Independent-Samples Mann-Whitney U Test	.285	Retain the null hypothesis.
16	The distribution of Albumin at admission is the same across categories of Delirium.	Independent-Samples Mann-Whitney U Test	.498	Retain the null hypothesis.
17	The distribution of Ammonia in the EDIMCU stay is the same across categories of Delirium.	Independent-Samples Mann-Whitney U Test	.931[1]	Retain the null hypothesis.
18	The distribution of Glucose at admission is the same across categories of Delirium.	Independent-Samples Mann-Whitney U Test	.088	Retain the null hypothesis.
19	The distribution of Blood urea nitrogen at admission is the same across categories of Delirium.	Independent-Samples Mann-Whitney U Test	.001	Reject the null hypothesis.
20	The distribution of Blood urea nitrogen at discharge is the same across categories of Delirium.	Independent-Samples Mann-Whitney U Test	.006	Reject the null hypothesis.

Asymptotic significances are displayed. The significance level is .05.

[1] Exact significance is displayed for this test.

Hypothesis Test Summary

	Null Hypothesis	Test	Sig.	Decision
21	The distribution of Creatinine at admission is the same across categories of Delirium.	Independent-Samples Mann-Whitney U Test	.006	Reject the null hypothesis.
22	The distribution of Creatinine at discharge is the same across categories of Delirium.	Independent-Samples Mann-Whitney U Test	.148	Retain the null hypothesis.
23	The distribution of Arterial pH at admission is the same across categories of Delirium.	Independent-Samples Mann-Whitney U Test	.264	Retain the null hypothesis.
24	The distribution of Arterial PaO2 at admission is the same across categories of Delirium.	Independent-Samples Mann-Whitney U Test	.865	Retain the null hypothesis.
25	The distribution of Arterial pCO2 at admission is the same across categories of Delirium.	Independent-Samples Mann-Whitney U Test	.458	Retain the null hypothesis.
26	The distribution of Arterial ionized calcium at admission is the same across categories of Delirium.	Independent-Samples Mann-Whitney U Test	.204	Retain the null hypothesis.
27	The distribution of Arterial ionized calcium at discharge is the same across categories of Delirium.	Independent-Samples Mann-Whitney U Test	.035	Reject the null hypothesis.
28	The distribution of Osmolarity is the same across categories of Delirium.	Independent-Samples Mann-Whitney U Test	.001	Reject the null hypothesis.

Asymptotic significances are displayed. The significance level is .05.

[1] Exact significance is displayed for this test.
